# RESHAPING LONG-TERM CARE SYSTEM INTO A SUSTAINABLE AND HIGH-QUALITY HEALTHCARE FIELD GLOBALLY: IT IS POSSIBLE!

**DOI:** 10.1093/geroni/igae098.1948

**Published:** 2024-12-31

**Authors:** Katherine McGilton, Karen Spilsbury, Carl Thompson

**Affiliations:** Chair; Co-Chair; Discussant

## Abstract

Years of investigation have revealed that despite compelling evidence, long-term care (LTC) home policies and procedures remain resistant to change. Globally, LTC homes grapple with subpar labor conditions stemming from structural and institutional elements, resulting in precarious work environments for its workforce. The revitalization of the LTC necessitates immediate attention to adequate staff and skill mix and innovative models of care to enable effective and person-centered care of residents. In this symposium, we will present an interactive overview and discussion of the work from international researchers exploring sustainable recruitment and retention of the LTC workforce, optimal staff and skill mix and initiatives for the intricate care requirements of residents and to mitigate precarious work conditions for the workforce. Specifically,: 1. Dr. Devi’s team presents findings from a Realist Review called the REACH including an explanatory framework to implement strategies to attract, recruit and retain LTC staff. 2. Dr. Montse and team highlight findings from a narrative review of measures taken by national European administrations to optimize working conditions for the LTC workforce including during the COVID-19 pandemic. 3. Dr. Bleijlevens and colleagues present the successful implementation of DAIly NURSE Intervention that transformed breakfast time for LTC residents. 4. Dr. McGilton and team implemented and evaluated the impact of nurse practitioner-led huddles that helped reduce moral distress amongst staff. 5. Prof. Zúñiga’s team prepares the scale-up of a registered nurse-led care model strengthening their geriatric expertise to reduce unplanned hospitalization for LTC residents.

